# Combination antiretroviral therapy prevents SIV- induced aging in the hippocampus and neurodegeneration throughout the brain

**DOI:** 10.21203/rs.3.rs-4681317/v1

**Published:** 2024-08-05

**Authors:** Andrew MacLean, Miranda Horn, Cecily Midkiff, Alison Van Zandt, Ahmad Saied

**Affiliations:** Tulane National Primate Research Center

**Keywords:** neuropathology, inflammaging, senescence, glia, aging, HIV, neurodegeneration

## Abstract

Virus-induced accelerated aging has been proposed as a potential mechanism underlying the persistence of HIV-associated neurocognitive disorders (HAND) despite advances in access and adherence to combination antiretroviral therapies (cART). While some studies have demonstrated evidence of accelerated aging in PLWH, studies examining acute infection, and cART intervention are limited, with most studies being *in vitro* or utilizing small animal models. Here, we utilized FFPE tissues from Simian immunodeficiency virus (SIV) infected rhesus macaques to assess the levels of two proteins commonly associated with aging - the cellular senescence marker p16^INK4a^ (p16) and the NAD-dependent deacetylase sirtuin 1 (SIRT1). Our central hypothesis was that SIV infection induces accelerated aging phenotypes in the brain characterized by increased expression of p16 and altered expression of SIRT1 that correlate with increased neurodegeneration, and that cART inhibits this process. We found that SIV infection induced increased GFAP, p16, SIRT1, and neurodegeneration in multiple brain regions, and treatment with cART reduced GFAP expression in SIV-infected animals and thus likely decreases inflammation in the brain. Importantly, cART reversed SIV-induced accelerated aging (p16 and SIRT1) and neurodegeneration in the frontal lobe and hippocampus. Combined, these data suggest that cART is both safe and effective in reducing neuroinflammation and age-associated alterations in astrocytes that contribute to neurodegeneration, providing possible therapeutic targets in the treatment of HAND.

## Introduction

The advent and use of combination antiretroviral therapies (cART) has greatly improved the lifespan of people living with HIV (PLWH) to near that of the HIV-negative population. However, the onset of age-related disorders in PLWH remains over a decade earlier. Within the brain, use of cART has drastically decreased the prevalence of the most severe form of HIV-associated neurocognitive disorders (HAND) – HIV-associated dementia, yet the prevalence of milder forms – Asymptomatic Neurocognitive Impairment and Mild Neurocognitive Disorder – has increased. Additionally, whole brain imaging and neurocognitive assessments indicate an accelerated aging phenotype in PLWH even with an undetectable viral load [1]. Whether cART contributes to neurodegeneration via direct neurotoxicity, incomplete suppression of viral replication in the CNS, or other mechanisms is yet to be determined [2].

An emerging theory is that chronic infection with HIV triggers an accelerated biological aging process. This theory is supported by multiple molecular markers of aging being altered throughout peripheral tissues of PLWH. For example, expression of the cellular senescence marker p16^INK4a^ (p16) in T cells correlated with chronological age in uninfected controls and HIV-infected, cART-treated individuals, but was significantly higher in HIV-infected untreated individuals, indicating an accelerated aging phenotype [3, 4]. Additionally, in a recent longitudinal study of PLWH, epigenetic aging in peripheral blood mononuclear cells was also found to accelerate with HIV infection and decelerate after successful viral suppression with cART [5], demonstrating an accelerated aging phenotype with HIV infection that is inhibited by cART. However, when looking at what is occurring within the CNS, support for accelerated biological aging is more limited, especially in the context of cART.

One study found that HIV infection in humans accelerated aging in the occipital lobe and cerebellum relative to uninfected controls based on DNA methylation measures; however, they did not assess the impact of cART on this process [6]. In HIV transgenic rats, p16 was elevated in both the frontal cortex and striatum in general, as well as in microglia specifically relative to wildtype animals [7]. The same group also previously found sirtuin 1 (SIRT1), a NAD-dependent deacetylase implicated in lifespan regulation, to be decreased at both the mRNA and protein level in the frontal cortex of HIV transgenic rats relative to wildtype animals [8]. Together, these studies support the theory that HIV accelerates aging in the brain; however, as these studies were carried out in transgenic rodents, the impact of cART on the aging process could not be assessed.

The effects of cART on molecular markers of biological aging in the brain have largely been restricted to *in vitro* studies. In human astrocyte cultures, exposure of cells to antiretroviral drugs has been associated with numerous signs of biological aging, including decreased proliferation, and increased expression of senescence-associated β-galactosidase and the cell-cycle arrest protein p21 [9]. As the penetrance of cART into the brain is limited, the effect of direct exposure to cART drugs may not be representative of what occurs *in vivo*. Thus, there is a critical need to assess the impact of cART on biological aging in the brain using *in vivo* models that allow for long-term cART administration.

Research with nonhuman primates (NHPs) is essential to understanding the pathogenesis of HIV – through application of the highly-translational Simian immunodeficiency virus (SIV) – and has been critical to assessing the efficacy of various therapeutic strategies, including cART [10]. Additionally, due to their genetic and physiological similarities to humans, NHPs are essential for studying the aging process, especially in the brain [11, 12]. Our lab recently found p16 increased with age in the frontal and temporal lobes and correlated with neurodegeneration in the frontal lobe and cerebellum of NHPs [13]. Additionally, SIRT1 expression increased with age in the temporal lobe and correlated with neurodegeneration in the frontal lobe. Given the importance of astrocytes in maintaining homeostasis in the brain, supporting neuronal function, and regulating the blood-brain barrier, we also assessed the expression of these markers in astrocytes specifically and found similar patterns, highlighting a potential role for astrocytes in eugeric aging. However, the impact of SIV infection and cART treatment on p16 and SIRT1 expression in the NHP brain has not previously been assessed, especially in astrocytes.

Based on these findings, the central hypothesis for this study was that exposure to SIV triggers an accelerated biological aging phenotype in the brain that is characterized by increased expression of p16 and altered expression of SIRT1 that correlate with increased neurodegeneration. Additionally, as cART eliminates productive viral replication, we expect treatment with cART to inhibit these alterations at least partially. To test these hypotheses, we utilized archival rhesus macaque tissues from multiple brain regions implicated in HAND and immunofluorescent staining techniques to assess the expression of p16, SIRT1, and the neurodegeneration marker FluoroJade C (FJC) as well as glial fibrillary acidic protein (GFAP) to assess the role astrocytes play in the process.

## Materials and Methods

### Ethics Statement

All animals used for this study were from previous studies at the Tulane National Primate Research Center (TNPRC; Covington, LA) and were handled in accordance with the American Association for Accreditation of Laboratory Animal Care and NIH “Principles of laboratory animal care”. All animal procedures were carried out by veterinarians and their staff as approved by the Institutional Animal Care and Use Committee of Tulane University. In accordance with endpoint policies, animals were humanely euthanized by veterinary staff by anesthesia with ketamine hydrochloride (10 mg/kg) followed by an overdose with sodium pentobarbital and necropsy. Tissues were collected and fixed for 48 hours in 10% neutral buffered formalin with zinc modification prior to being embedded in paraffin.

### Selection of Animals and Tissues

This study utilized archival formalin-fixed paraffin-embedded (FFPE) tissues from 18 rhesus macaques. Animals ranged in age from 4 to 15 years of age and both males and females were included. However, due to nonhuman primates being an acutely scarce resource and breeding-aged females rarely being used in studies, we were unable to balance sexes in the groups. Animals were selected based on infection status with SIVmac239 or SIVmac251, treatment with a cART regimen of Tenofovir Disoproxil Fumarate (TDF) 5.1 mg/kg, Emtricitabine (FTC) 30 mg/kg, and Dolutegravir (DTG) 2.5 mg/kg, and availability of appropriate histological brain sections. Based on these criteria, we identified three animals that were naïve to SIV and cART and were healthy at the time of investigator-initiated euthanasia (Naïve), three animals that had been infected with SIV for only 21 days (Acute SIV), five animals that had been infected with SIV for greater than 55 days without receiving cART (Chronic SIV), and seven animals that had been infected with SIV for 77–91 days prior to the initiation of a daily cART regimen until the time of euthanasia (SIV-cART). For more detailed information on the animals used in this study, see Table 1.

Previous imaging studies have identified several brain regions that show atrophy in PLWH and SIV-infected macaques, including the frontal lobe, hippocampus, caudate, putamen, thalamus, and cerebellum [14–18]. Based on these findings, we used H&E slides to select FFPE tissue blocks that contained these brain regions for each animal whenever possible. Some animals did not have samples for each of the brain regions listed above, thus the number of tissues analyzed for each brain region varies. See Table 1 for the specific regions analyzed for each animal.

### Immunohistochemistry

For analysis of microglia/macrophages, chromogenic immunohistochemistry for Iba1 was performed by the TNPRC Confocal Microscopy and Molecular Pathology Core. Tissues were cut at 5μm and mounted on charged slides, then baked for at least three hours at 60°C prior to being passed through xylene, graded ethanol, and double distilled water to remove paraffin and rehydrate tissue sections. Next, slides were boiled for 16 minutes in a Tris based solution, pH 9 (Vector Labs H-3301), containing 0.1% Tween20 before being briefly rinsed in hot, deionized water and transferred to a hot citrate-based solution, pH 6.0 (Vector Labs H-3300) where they were allowed to cool to room temperature. Slides were removed from the antigen retrieval solution, washed in phosphate-buffered saline, deionized water, and Roche reaction buffer before being loaded on the Ventana Discovery Ultra autostainer where they would undergo blocking, primary antibody (rabbit anti-IBA, Wako 019–19741, 1:3000 dilution) incubation, washing, secondary antibody incubation (OMap anti-Rb HRP, Ventana, cat. #760–4311), washing, DAB color development, and counterstaining with hematoxylin II. Upon removal, slides were put through alternating manual washes of deionized water containing 0.1% Dawn dish soap and plain deionized water for a total of 5 cycles. Slides were then cleared in ethanol (80%, 95%, 100%, 100%) and three xylene changes before being permanently mounted with StatLab^™^ AcryMount Plus Mounting Media (FisherScientific, cat. # STSL80PLUS4) and left overnight to dry prior to imaging.

### Immunofluorescent Staining

For all immunohistochemistry, formalin-fixed paraffin-embedded tissues were cut at a thickness of 5 and placed onto charged slides. Slides were baked at 60°C and deparaffinized by incubating three times for 5 minutes in xylene and rehydrating in decreasing concentrations of alcohol (100% twice for 3.5 minutes, 95% once for 3 minutes, and 80% once for 5 minutes). For p16^INK4a^ (anti-p16^INK4a^, Invitrogen, Ms Mab IgG1, MA5–17093, 1:100) and SIRT1 (anti-SIRT1, Lifespan Biosciences, Inc., Gt Pab, cat. #LS-B8356, 1:100), slides were rinsed in water then antigen retrieval was performed by bringing antigen-retrieval solution (Antigen Unmasking Solution, Citric Acid Based, Vector Laboratories, Inc., cat. #H-3300) to a boil, then incubating the slides in the solution uncovered for one hour. Non-specific binding was blocked by application of normal serum [normal goat (MP Biomedicals, LLC., cat. #2939149) or normal donkey (GeminiBio, cat. #100–151) serum] for 40 minutes prior to the application of primary antibodies. Anti-p16^INK4a^ was applied for 1 hour at room temperature while anti-SIRT1 was applied overnight at 4°C. Secondary antibodies and conjugated cell-specific antibodies (Goat anti-Mouse IgG (H + L), Alexa Fluor^™^ 633, Invitrogen, cat. #A-21052; Donkey anti-Goat IgG (H + L) Cross-Adsorbed Secondary Antibody, Alexa Fluor^™^ 568, Invitrogen, cat. #A-11057; Anti-Glial Fibrillary Acidic Protein (GFAP) – Cy3^™^ antibody, Sigma-Aldrich^®^, Ms Mab, cat. #C9205, 1:300; and GFAP Monoclonal Antibody (GA5), Alexa Fluor^™^ 488, eBioscience^™^, Ms Mab, cat. #53–9892-82, 1:300) were applied for 1 hour at room temperature. Slides were washed twice in PBS-FSG-Tx100 (1X Phosphate-Buffered Solution diluted from 10X Phosphate-Buffered Solution, Fisher BioReagents^™^, cat. #BP 3994; 0.2% Fish-Skin Gelatin, Sigma Aldrich, cat. #1002923460; 0.1% Triton-100, Fisher BioReagents^™^, cat. #BP 151 500) for 5 minutes and once in 1X PBS-FSG for 5 minutes after incubation with each antibody. Slides were washed for 5 minutes in 1X PBS prior to applying mounting media (EverBrite TrueBlack^®^ Hardset Mounting Medium, Biotium, cat. #23018) and coverslips.

### FluoroJade C staining

For FJC (Fluoro-Jade C Staining Kit with DAPI Counter Stain, Histo-Chem Inc., cat. #FJC-SK-DAPI) staining, following the deparaffinization described above, slides were incubated in 70% alcohol for 2 minutes followed by deionized water for 2 minutes. Autofluorescence was dampened by incubating slides in potassium permanganate for 5 minutes. Slides were then washed twice in deionized water for 2 minutes and placed in the FJC and DAPI solution for 10 minutes. Slides were then washed three times in deionized water for 1 minute and dried in the oven for 15 minutes at 60°C. Finally, slides were cleared in xylene for 1 minute prior to application of mounting media (Micromount mounting media, Leica Biosystems, cat. #3801730) and coverslips. After applying coverslips, slides were allowed to set at room temperature overnight before scanning.

### Image Analysis

For GFAP, p16, and SIRT1 analyses, slides were scanned using a Zeiss AxioScan.Z1 slide scanner at 20x magnification. Scan settings were optimized for each brain region and stain and the same scan profile was used across all slides for a given brain region and stain. Scanned images were loaded into the HALO^®^ Image Analysis software (Indica Labs) for analysis. Images were annotated for appropriate regions of interest and then parameters were set using the Highplex FL module (Indica Labs) to identify phenotypes of interest. Finally, whole-slide images and regions of interest were analyzed. Data were exported to Microsoft Excel where the percentage of marker-positive cells, dual-labeled cells, and relative fluorescent intensity for each marker were extracted and organized for statistical analysis. For Iba1 and microglia activation analyses, slides were scanned using a Hamamatsu NanoZoomer360 at 40x magnification. Images were loaded into the HALO^®^ image analysis software and analyzed using optimized settings in the Microglia Activation Module for each brain region to determine the percentage of Iba1 + cells and activated Iba1 + cells.

### Statistical Analyses

To assess the significance of differences in marker expression between groups, the percentage of marker-positive cells, dual-labeled cells, and relative intensity for each group were compared using a one-way ANOVA and Tukey’s post-hoc test in Prism GraphPad (version 10, GraphPad Software, La Jolla, CA). To determine the relationship between expression of multiple markers, two-tailed Pearson’s correlation coefficients were calculated using Prism GraphPad. Finally, to determine if p16 and SIRT1 expression predict neurodegeneration, separate multiple linear regressions were performed with %FJC + cells as the dependent variable or FJC intensity as the dependent variable. A p value < 0.05 was considered significant for all analyses.

## Results

### Description of pathology from selected animals

**Figure 1:**
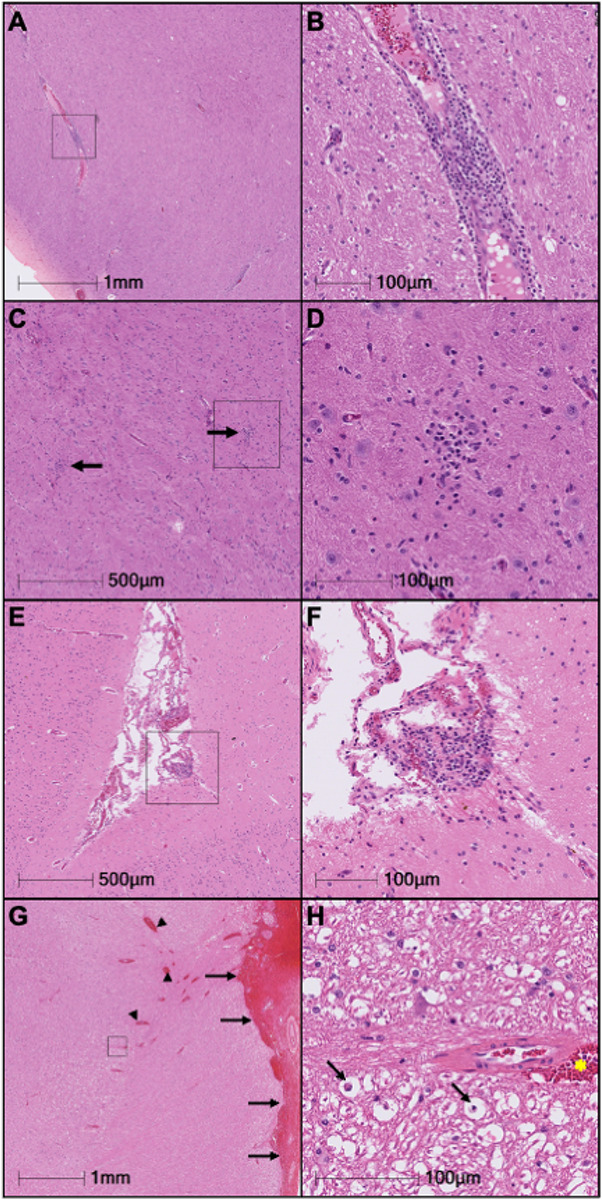
Representative histopathology of the brain. (A-D): Images from FV53 (Chronic SIV). (A) Virchow-Robin spaces are infiltrated by small to moderate numbers of lymphocytes and plasma cells. (B) Higher magnification of the reference frame from panel A. (C) Thalamus, multifocal glial nodules. (D) Higher magnification of the reference frame from panel C. (E-F): Images from MV13 (Acute SIV). (E) The meninges are focally infiltrated by a small number of mononuclear inflammatory cells. (F) Higher magnification of the reference frame from panel E. (G-H): Images from EI69 (Chronic SIV). (G) The meninges (black arrows) and Virchow Robin spaces (arrow heads) are markedly expanded by hemorrhage. (H) Higher magnification of the reference frame from panel A showing hemorrhage in a Virchow Robin space (yellow asterisk), and axonal degeneration and axonophagia (black arrows).

Animals infected with SIV in this study developed perivascular inflammation, meningitis, and microglial nodules in the brain. The histological changes in the brains of chronically infected animals were more pronounced than that of the acutely infected animals. Detailed examination revealed minimal inflammation in the meninges and choroid plexus of acutely infected animals (Table 1), moderate inflammation in the chronically infected without cART, and minor inflammation in the cART treated SIV-infected animals (Fig. 1). Consistent with these findings, the percentage of Iba1 + cells were elevated in chronically infected animals compared to SIV-cART animals in multiple brain regions but no other significant differences in the percentage of Iba1 + cells were observed (Supplemental Fig. 1).

### GFAP expression increases with SIV infection in a region-specific manner

**Figure 2:**
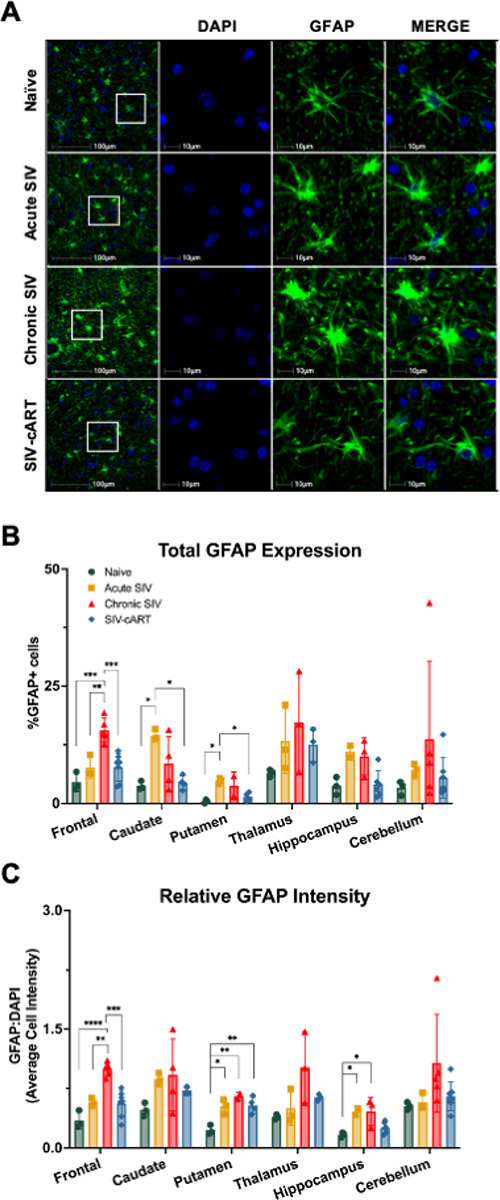
Treatment with cART reduces SIV-induced elevation of GFAP+ and Iba1+ cells in multiple brain regions of rhesus macaques. (A) Representative immunofluorescent images of GFAP staining in the white matter of the frontal lobe. (B) The percentage of GFAP+ cells was significantly elevated in the frontal lobe of chronic SIV-infected animals and in the caudate and putamen of acutely infected animals relative to naïve and cART treated animals. (C) The average cell intensity of GFAP staining relative to that of DAPI staining was also significantly increased in the frontal lobe of chronic SIV-infected animals as compared to all other groups, in the putamen of all SIV-infected animals regardless of cART, and in the hippocampus of SIV-infected, untreated animals. All data points are presented and mean +/− SD are plotted for each group. Each brain region was analyzed independently using a one-way ANOVA and Tukey’s post-hoc test. * p < 0.05, ** p <0.01, *** p < 0.001, **** p < 0.0001.

To assess the role of astrocytes in SIV-induced neurodegeneration, we first wanted to assess changes in astrocyte activation across all six brain regions. We expected to see increased astrocyte activation, as measured by increased expression of GFAP, in both acutely and chronically SIV-infected animals across all brain regions. To test this hypothesis, we stained slides for DAPI (blue) and GFAP (green) and assessed the percentage of co-labeled cells (Fig. 2). We found the percentage of GFAP + cells increased with chronic SIV infection in the frontal lobe (Fig. 2b, p = 0.0002) and with acute SIV infection in the caudate and putamen (Fig. 2b, p = 0.0114 and p = 0.0118). However, changes in the percentage of GFAP + cells with SIV infection in the thalamus, hippocampus, or cerebellum did not reach statistical significance.

Recently, we found that increased GFAP intensity better correlated with increasing age than the percentage of GFAP-positive cells [13]. Therefore, we hypothesized that if SIV infection increases aging it would increase GFAP intensity as well. When assessing GFAP intensity relative to DAPI intensity, we found GFAP to increase with chronic SIV infection in the frontal lobe, putamen, and hippocampus (Fig. 2c, p < 0.0001, p = 0.0025, and p = 0.0178, respectively). We also found GFAP intensity was increased with acute infection in the putamen and hippocampus (Fig. 2c, p = 0.0112 and p = 0.0346, respectively). This data suggests that astrocytes in these regions may be more sensitive to infection with SIV or more directly impacted than astrocytes in the caudate, thalamus, and cerebellum.

To our knowledge, the effect of long-term cART on GFAP expression has not previously been assessed *in vivo*. Here, we show that treatment with cART significantly decreased both the percentage of GFAP + cells and relative intensity of GFAP from that of chronically SIV-infected animals in the frontal lobe (Fig. 2b, p = 0.0007; Fig. 2c, p = 0.0002). Additionally, cART-treated animals had a significantly lower percentage of GFAP + cells than acutely infected animals in the caudate and putamen (Fig. 2b, p = 0.0111 and p = 0.0315, respectively). Importantly, the only place we observed a significant increase in GFAP intensity in cART-treated animals relative to naïve animals was in the putamen (Fig. 2c, p = 0.0058). Overall, it appears as though treatment with cART reduces GFAP expression in SIV-infected animals and thus likely decreases inflammation in the brain.

### p16INK4a expression increases with SIV infection across all brain regions

We have previously shown p16 increases with age in rhesus macaques, especially in the frontal lobe [13], thus we expected to see an SIV-induced increase in p16 throughout the selected brain regions indicative of premature or accelerated aging. To test this hypothesis, we stained slides from each brain region for DAPI, GFAP, and p16 (red), obtained whole-slide images, and assessed the percentage of p16 + cells in the total cell population and in the GFAP + cell population, as well as the relative intensity of p16.

**Figure 3:**
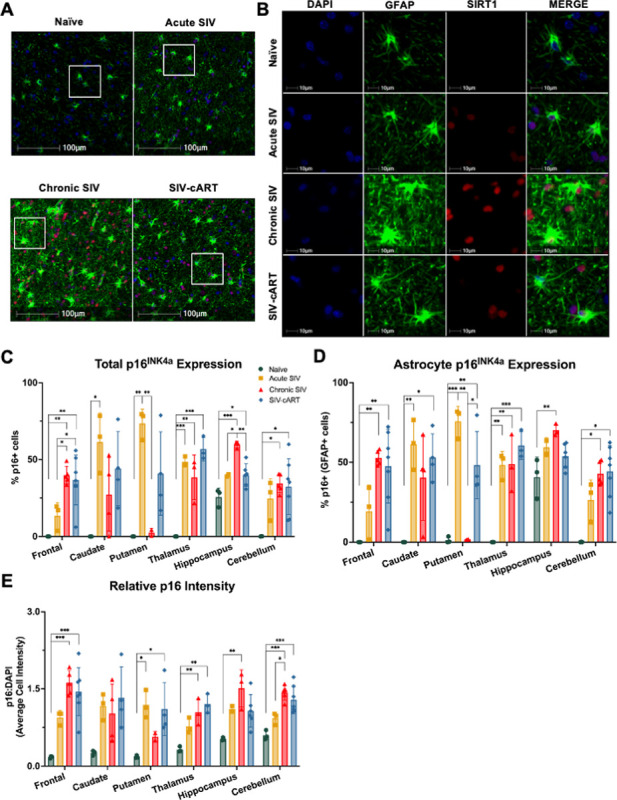
p16^INK4a^ expression is elevated across multiple brain regions in SIV-infected rhesus macaques. (A) Representative immunofluorescent images of p16 staining across groups in the white matter of the frontal lobe (B) The percentage of p16+ cells is elevated in SIV-infected animals relative to naïve animals in each brain region. (C) The percentage of p16+ astrocytes is elevated in SIV-infected animals relative to naïve animals in all brain regions. (D) The relative intensity of p16 staining is elevated in SIV-infected animals relative to naïve animals in all brain regions, except the caudate. All data points are presented and mean +/− SD are plotted for each group. Each brain region was analyzed independently using a one-way ANOVA and Tukey’s post-hoc test. * p < 0.05, ** p <0.01, *** p < 0.001.

Relative to young, naïve rhesus macaques (4–5 years of age), where there was minimal expression of the senescence marker, we observed robust increases in p16 expression with SIV infection across all brain regions (Fig. 3). In the caudate, putamen, and thalamus, we saw significant increases in the total percentage of p16 + cells after acute infection (Fig. 3b, p = 0.0182, p = 0.0036, and p = 0.0005, respectively), while for the frontal lobe, hippocampus, and cerebellum chronic infection was necessary to observe significant increases in the percentage of p16 + cells relative to naïve animals (Fig. 3b, p = 0.0015, p = 0.0002, and p = 0.0147, respectively). Additionally, in the frontal lobe and hippocampus, there was a significant increase in the percentage of p16 + cells between acute and chronic SIV infection (Fig. 3b, p = 0.0318 and p = 0.0170, respectively), suggesting a progressive effect in these regions. Interestingly, the increase in total percentage of p16 + cells seen with acute infection was lost with chronic infection in the caudate and putamen but remained in the thalamus (Fig. 3b, p = 0.0022). This may be due in part to the low number of tissues available from chronically infected animals for the caudate and putamen analyses. It is also important to note that only in the hippocampus did treatment with cART significantly reduce the percentage of p16 + cells from that in chronically SIV-infected animals (Fig. 3b, p = 0.0035), though levels were still significantly higher than in naïve animals (p = 0.0276). The percentage of p16 + cells was also significantly elevated in the frontal lobe, thalamus, and cerebellum of cART-treated animals relative to naïve animals (p = 0.0019, p = 0.0001, and p = 0.0151, respectively) and relative to acutely infected animals in the frontal lobe (p = 0.0474). This suggests that while cART was effective in reducing productive viral infection peripherally, it did not significantly alter the expression of p16 following chronic SIV-infection in the brain.

When looking at the percentage of astrocytes that were p16+, we saw a similar pattern (Fig. 3c). Again, we see that acute SIV infection in the caudate, putamen, and thalamus is sufficient to trigger a significant increase in the percentage of p16 + cells (Fig. 3c, p = 0.0092, p = 0.0008, and p = 0.0025, respectively). Again, this elevation is lost with chronic infection in the caudate and putamen but remains significant in the thalamus (p = 0.0024). We also saw significant increases in the percentage of p16 + astrocytes in the frontal lobe, hippocampus, and cerebellum of chronically infected animals (p = 0.0031, p = 0.0061, and p = 0.0015, respectively). The percentage of p16 + astrocytes was also significantly elevated in cART-treated animals across the frontal lobe, caudate, putamen, thalamus, and cerebellum (p = 0.0046, p = 0.0149, p = 0.0092, p = 0.0006, and p = 0.0007, respectively) and was not decreased in any brain region relative to that in chronically infected animals. Again, this suggests that cART does not significantly alter the expression of p16 in the brain from that seen in chronically infected animals.

To assess changes in the overall expression of p16, we analyzed the intensity of p16 staining relative to that of DAPI and again saw a similar pattern of increased p16 expression with SIV infection across the brain (Fig. 3d). One difference, however, was that with acute infection we only saw a significant increase in p16 expression in the putamen (p = 0.0003) and not in the caudate or thalamus. With chronic infection, we saw a significant increase in p16 expression in the frontal lobe, thalamus, hippocampus, and cerebellum (p = 0.0002, p = 0.0047, p = 0.0065, p = 0.0003, respectively). We also saw a significant increase in p16 expression in cART-treated animals relative to naïve animals in the frontal lobe, putamen, thalamus, and cerebellum (p = 0.0004, p = 0.0328, p = 0. 0014, p = 0.0010, respectively), with no significant reductions relative to SIV-infected, untreated animals. Thus, cART again appears to neither increase nor decrease p16 expression from that seen in untreated, SIV-infected animals.

### SIRT1 expression varies based on brain region and infection status

The second aging marker we assessed was SIRT1. We previously found a positive correlation between SIRT1 expression and neurodegeneration in the frontal lobe of uninfected animals [13]. Additionally, it is known that the activity of SIRT1 is inhibited by the HIV protein Tat leading to hyperactivation of immune cells [19]. Thus, we were interested in how these interactions may impact expression in the brain, especially in astrocytes. Based on the reduction of SIRT1 at the mRNA and protein levels in the gut of SIV-infected animals [20], we expected to see similar reductions in the brain. To test this hypothesis, we stained slides for DAPI, GFAP, and SIRT1 (red), then obtained whole-slide scanned images and assessed the percentage of SIRT1 + cells, the percentage of SIRT1 + astrocytes, and the relative intensity of SIRT1 staining.

**Figure 4:**
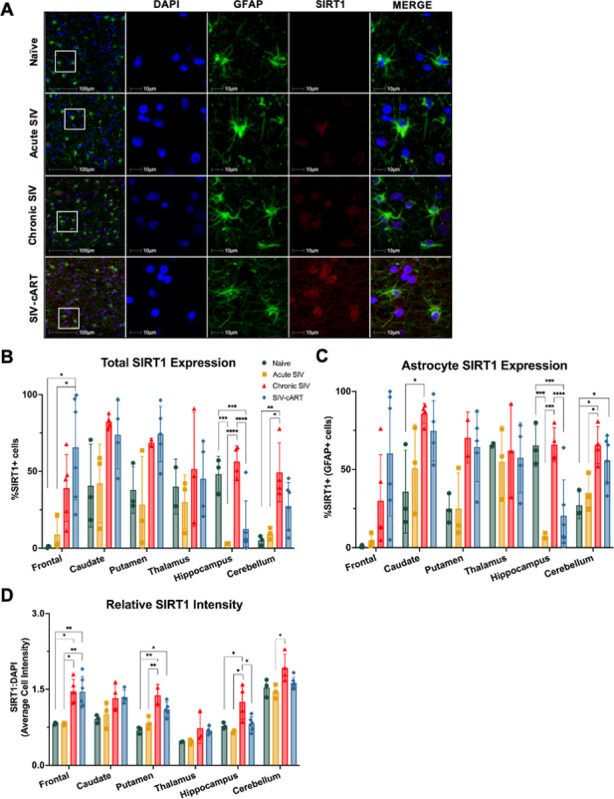
Expression of SIRT1 increases with SIV infection in several brain regions of the rhesus macaque. (A) Representative immunofluorescent images of SIRT1 staining across groups in the white matter of the frontal lobe. (B) Relative to naïve animals, the percentage of SIRT1+ cells was elevated in the frontal lobe of cART-treated animals and cerebellum of chronic SIV animals but reduced in the hippocampus of acutely infected animals and cART-treated animals. (C) Relative to naïve animals, the percentage of SIRT1+ astrocytes was elevated in the caudate of chronic SIV animals and in the cerebellum of chronic SIV animals and cART-treated animals but reduced in the hippocampus of acutely infected animals and cART-treated animals. (D) The average cell intensity of SIRT1 staining relative to DAPI was elevated in the frontal lobe, putamen, hippocampus, and cerebellum of chronic SIV animals and in the frontal lobe and putamen of cART-treated animals. In the hippocampus, cART-treated animals had significantly reduced SIRT1 staining relative to chronic, untreated animals. All data points are presented and mean +/− SD are plotted for each group. Each brain region was analyzed independently using a one-way ANOVA and Tukey’s post-hoc test. * p < 0.05, ** p <0.01, *** p < 0.001, **** p < 0.0001.

In general, the relationship between SIV infection and SIRT1 expression appears to be region specific even in naïve animals. When looking at the percentage of SIRT1 + cells, we saw a significant reduction relative to naïve animals in the hippocampus of acutely infected animals (Fig. 4b, p = 0.0005) that rebounded with chronic infection (p < 0.0001). In the cerebellum, we saw a significant increase in the percentage of SIRT1 + cells with chronic infection, relative to both naïve animals (p = 0.0063) and acutely infected animals (p = 0.0132). In the frontal lobe, caudate, putamen, and thalamus, we did not see any significant changes in the percentage of SIRT1 + cells with acute or chronic infection. Finally, cART had opposing effects in the frontal lobe and hippocampus. In the frontal lobe, we saw a significant increase in SIRT1 + cells in cART-treated animals relative to both naïve animals (p = 0.0128) and acutely infected animals (p = 0.0291). Whereas in the hippocampus, cART-treated animals had a significant reduction in SIRT1 + cells relative to naïve animals (p = 0.0001) and chronically infected animals (p < 0.0001) that more closely resembled levels seen in acutely infected animals.

Next, we assessed the effect of SIV infection on expression of SIRT1 in astrocytes specifically (Fig. 4c). Here, we saw a similar, region-specific effect. Similar to the total cell population, we saw a significant reduction in the percentage of SIRT1 + astrocytes in the hippocampus of acutely infected animals (p = 0.0006) that rebounded with chronic infection (p = 0.0004). Whereas we saw a significant increase in SIRT1 + astrocytes in chronically infected animals relative to naïve animals in the caudate (p = 0.0412) and cerebellum (p = 0.0109), with the increase in the cerebellum also being significantly higher than that in acutely infected animals (p = 0.0435). Finally, cART-treated animals had significantly reduced SIRT1 + astrocytes in the hippocampus relative to both naïve animals (p = 0.0002) and chronically infected animals (p< 0.0001) that resembled levels seen in acutely infected animals. However, cART-treated animals had elevated SIRT1 + astrocytes in the cerebellum relative to naïve animals (p = 0.0396).

To better understand how SIRT1 expression is altered with SIV infection, we also analyzed the intensity of SIRT1 staining relative to DAPI staining (Fig. 4d). Here, we saw more consistent patterns across the brain. To begin, there were no significant differences in SIRT1 expression between naïve and acutely infected animals in all brain region examined. Chronically infected animals had a significant increase in SIRT1 expression relative to naïve animals in the frontal lobe (p = 0.0104), putamen (p = 0.0018), and hippocampus (p = 0.0453) and relative to acutely infected animals in the frontal lobe (p = 0.0101), putamen (p = 0.0083), hippocampus (p = 0.0279), and cerebellum (p = 0.0215). Animals treated with cART also had increased SIRT1 expression relative to naïve animals in the frontal lobe (p = 0.0089) and putamen (p = 0.0117). Whereas in the hippocampus, SIRT1 expression was significantly reduced in cART-treated animals relative to chronically infected animals (p = 0.0296) to levels that resemble that seen in naïve and acutely infected animals.

### FluoroJade C staining is increased in chronically SIV-infected animals in a region-specific manner

**Figure 5:**
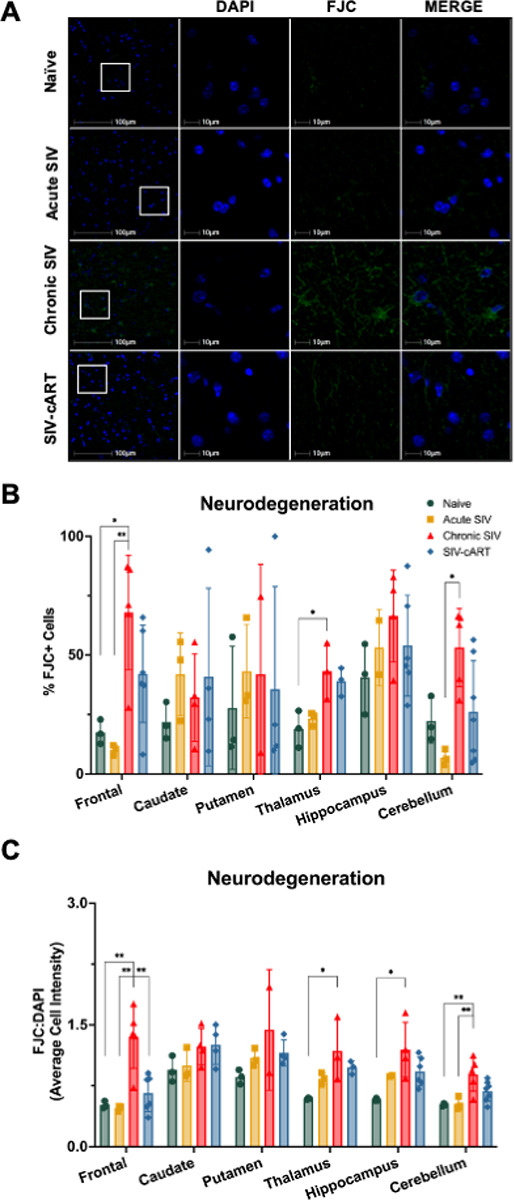
Untreated chronic SIV infection induces neurodegeneration in multiple brain regions of the rhesus macaque. (A) Representative immunofluorescent images of FJC staining across groups in the white matter of the frontal lobe. (B) The percentage of FJC+ cells is elevated in the frontal lobe, thalamus, and cerebellum of chronic SIV animals. (C) The average cell intensity of FJC staining relative to DAPI staining is elevated in the frontal lobe, thalamus, hippocampus, and cerebellum of chronic SIV animals. Treatment with cART significantly reduces FJC staining in the frontal lobe from that seen in chronic SIV animals. All data points are presented and mean +/− SD are plotted for each group. Each brain region was analyzed independently using a one-way ANOVA and Tukey’s post-hoc test. * p < 0.05, ** p <0.01.

To assess generalized neurodegeneration, we performed FluoroJade C (FJC) staining on all tissues, followed again by whole-slide scanning and analyses using HALO^®^ image analysis software. In general, we saw an increase in neurodegeneration, as measured by the percentage of FJC + cells or relative intensity of FJC staining, in chronically SIV-infected animals across several brain regions relative to naïve animals, but not in any other groups (Fig. 5). More specifically, when looking at the percentage of FJC + cells (Fig. 5b), we saw a significant increase relative to naïve animals in the frontal lobe (p = 0.0118) and thalamus (p = 0.0210) and a significant increase relative to acutely infected animals in the frontal lobe (p = 0.0044) and the cerebellum (p = 0.0104). When looking at the relative intensity of FJC staining, we saw a similar pattern (Fig. 5c). Relative FJC intensity increased with chronic infection relative to naïve animals again in the frontal lobe (p = 0.0031) and thalamus (p = 0.0285), as well as in the hippocampus (p = 0.0139) and cerebellum (p = 0.0131). Relative to acutely infected animals, chronically infected animals had elevated FJC staining again in the frontal lobe (p = 0.0021) and cerebellum (p = 0.0167). Interestingly, when using relative intensity of FJC as a measure of general neurodegeneration, cART-treated animals had significantly less neurodegeneration in the frontal lobe than chronically infected animals (p = 0.0031), suggesting a potential protective effect of cART in the frontal lobe. It is also important to note that neurodegeneration was not significantly elevated in cART-treated animals relative to naïve animals in any brain region by either measure used, and therefore does not appear to be eliciting additional neurodegeneration in the brain.

### Neurodegeneration correlates with markers of accelerated aging

**Figure 6:**
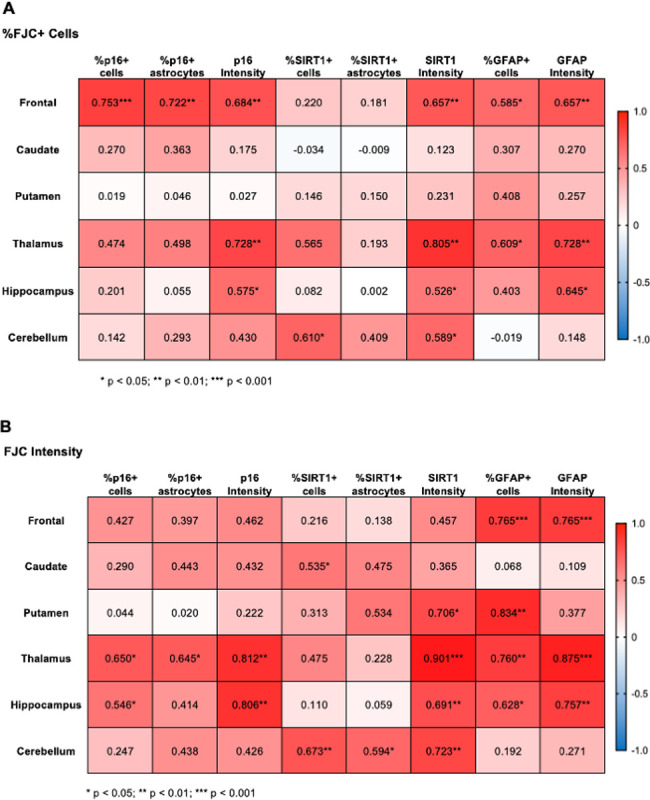
Expression of aging markers and GFAP positively correlate with neurodegeneration. (A) Correlations between aging markers, GFAP, and the percentage of FJC+ cells. (B) Correlations between aging markers, GFAP, and the average cell intensity of FJC staining relative to DAPI staining. Values presented are Pearson’s correlation coefficients with two-tailed p values. * p < 0.05, ** p <0.01, *** p < 0.001.

Finally, to assess the relevance of changes in p16 and SIRT1 expression to neurodegeneration, we performed Pearson correlation analyses between each marker and neurodegeneration (Fig. 6). Here we found a great deal of variability from one brain region to the next and depending on the measure of neurodegeneration used. When assessing the correlation of aging markers with the percentage of FJC + cells (Fig. 6a), the %p16 + cells and %p16 + astrocytes were only significantly correlated in the frontal lobe (p = 0.0005 and p = 0.001, respectively). However, p16 intensity correlated with %FJC + cells in the frontal lobe (p = 0.002), thalamus (p = 0.007), and hippocampus (p = 0.031). The %SIRT1 + cells only correlated with %FJC + cells in the cerebellum (p = 0.012) and the %SIRT1 + astrocytes did not correlate with %FJC + cells in any brain region. Yet SIRT1 intensity correlated with %FJC + cells in the frontal lobe (p = 0.004), thalamus (p = 0.003), hippocampus (p = 0.044), and cerebellum (p = 0.016). Finally, the %GFAP + cells correlated with %FJC + cells in both the frontal lobe (p = 0.014) and thalamus (p = 0.036), and the intensity of GFAP staining correlated with %FJC + cells in the frontal lobe (p = 0.004), thalamus (p = 0.007), and hippocampus (p = 0.013). When assessing the correlation of aging markers with the intensity of FJC staining (Fig. 6b), the %p16 + cells and p16 intensity were both significantly correlated in the thalamus (p = 0.022 and p = 0.001, respectively) and hippocampus (p = 0.043 and p = 0.001, respectively). The %p16 + astrocytes was also significantly increased in the thalamus (p = 0.023). The %SIRT1 + cells correlated with FJC intensity in the caudate (p = 0.049) and cerebellum (p = 0.004), while %SIRT1 + astrocytes was correlated with FJC intensity in the cerebellum only (p = 0.015). However, the intensity of SIRT1 staining correlated with FJC intensity in several brain regions, including the putamen (p = 0.010), thalamus (p = 0.00015), hippocampus (p = 0.004), and cerebellum (p = 0.002). Finally, the %GFAP + cells and GFAP intensity both correlated with FJC intensity in the frontal lobe (p = 0.0003454 and p = 0.0003499, respectively), thalamus (p = 0.004 and p = 0.00019, respectively), and hippocampus (p = 0.012 and p = 0.002, respectively), while only %GFAP + cells correlated with FJC intensity in the putamen (p = 0.001).

To determine if p16 and SIRT1 significantly predicted neurodegeneration, multiple linear regression was used. Here, we found both p16 expression and SIRT1 expression to predict FJC expression, but only in the frontal lobe and hippocampus. For the frontal lobe, when assessing if %p16 + cells and %SIRT1 + cells predicted %FJC + cells, the overall regression was significant (R^2^ = 0.6152, F(3, 13) = 6.927, p = 0.0050) and the %p16 + cells significantly predicted %FJC + cells (ß = 1.403, p = 0.0024). For the frontal lobe, when assessing if %p16 + astrocytes and %SIRT1 + astrocytes predicted %FJC + cells, the overall regression was significant (R^2^ = 0.5482, F(3, 13) = 5.258, p = 0.0135) and the %p16 + astrocytes significantly predicted %FJC + cells (ß = 0.9245, p = 0.0053). In the hippocampus, when assessing if %p16 + cells and %SIRT1 + cells predicted %FJC + cells, the overall regression was significant (R^2^ = 0.5279, F(3, 10) = 3.727, p = 0.0494) and the %p16 + cells significantly predicted %FJC + cells (ß = −2.545, p = 0.0236), as did the %SIRT1 + cells (ß = −2.718, p = 0.0104), and the interaction between %p16 + cells and %SIRT1 + cells (ß = 0.06323, p = 0.0093). Similarly for the hippocampus, when assessing if %p16 + astrocytes and %SIRT1 + astrocytes predicted %FJC + cells, the overall regression was significant (R^2^ = 0.7416, F(3, 10) = 9.567, p = 0.0028) and the %p16 + astrocytes significantly predicted %FJC + cells (ß = −3.258, p = 0.0007), as did the %SIRT1 + astrocytes (ß = −3.671, p = 0.0003), and the interaction between %p16 + astrocytes and %SIRT1 + astrocytes (ß = 0.01218, p = 0.0003). We saw similar results in the hippocampus when assessing if %p16 + cells and %SIRT1 + cells predicted FJC intensity, where the overall regression was significant (R^2^ = 0.6871, F(3, 10) = 7.321, p = 0.0070) and the %SIRT1 + cells significantly predicted FJC intensity (ß = −0.03660, p = 0.0057), as did the interaction between %p16 + cells and %SIRT1 + cells (ß = 0.0008359, p = 0.0056). Similarly for the hippocampus, when assessing if %p16 + astrocytes and %SIRT1 + astrocytes predicted FJC intensity, the overall regression was significant (R^2^ = 0.6141, F(3, 10) = 5.306, p = 0.0191) and the %p16 + astrocytes significantly predicted FJC intensity (ß = −0.02875, p = 0.0414), as did the %SIRT1 + astrocytes (ß = −0.041492, p = 0.0075), and the interaction between %p16 + astrocytes and %SIRT1 + astrocytes (ß = 0.0007491, p = 0.0069). Together, these results demonstrate the importance of these aging markers in SIV-induced neurodegeneration in the frontal lobe and hippocampus.

## Discussion

In this study, utilizing archival rhesus macaque tissues from six brain regions implicated in HAND, we found significant alterations of the aging markers p16 and SIRT1 in both the total cell population and in astrocytes with SIV infection that correlated with neurodegeneration. The astrocyte marker GFAP increased with infection in several brain regions, but was broadly decreased with cART treatment, suggestive of a protective effect of cART (Fig. 2). Expression of p16 was significantly elevated with SIV infection across all six brain regions for both the total cell population and astrocytes specifically (Fig. 3). Similarly, expression of SIRT1 was elevated with SIV infection in several brain regions, but the percentage of SIRT1 + cells decreased in the hippocampus with acute infection, suggesting a more region- and disease-specific response for SIRT1 expression (Fig. 4). Importantly, cART treatment only led to significant decreases in p16 and SIRT1 expression relative to chronic, untreated infection in the hippocampus, with significant increases relative to naïve animals in several brain regions (Figs. 4 and 5). Additionally, neurodegeneration significantly increased in chronically infected, untreated animals relative to naïve or acutely infected animals across several brain regions and treatment with cART significantly decreased neurodegeneration relative to chronically infected animals in the frontal lobe (Fig. 5). Furthermore, the expression of p16 and SIRT1 correlated with neurodegeneration in both the total cell population and in astrocytes across several brain regions (Fig. 6). Together, these data provide critical new insights into SIV-induced, aging-related changes throughout the brain.

While GFAP expression is widely used as a marker of astrocyte reactivity or astrogliosis, it has also been shown to increase with age. In a recent study of eugeric aging in the rhesus macaque brain, we found that while the percentage of GFAP + cells did not correlate with age, the intensity of GFAP staining was correlated with age in multiple brain regions [13]. Consistent with these findings, expression of GFAP has also been shown to increase with age across multiple regions of the brain in both rodents and humans [21, 22]. Additionally, the size and morphology of astrocytes have been shown to change with age in humans and NHPs [12, 23, 24], consistent with an increase in GFAP expression but not necessarily an increase in the number of GFAP + cells. This is of particular interest here as the intensity of GFAP, but not the percentage of GFAP + cells, increased with SIV infection in the putamen and hippocampus. Combined with the results for p16 and SIRT1, these results indicate a potential sensitivity of these regions to the aging effects of chronic infection with SIV.

As one of the most prominent markers of cellular senescence, p16 has been associated with aging in many species and cell types. Within the brain, p16 has been shown to increase with normal aging in the frontal cortex of humans [25] and in astrocytes specifically [26]. We also found p16 to increase with normal aging in astrocytes of the frontal and temporal lobes of rhesus macaques but not in the parietal lobe or cerebellum [13]. Thus, p16 may increase with age in a region-specific manner. In the context of HIV, p16 has been shown to increase in the frontal lobe of PLWH and in microglia of HIV-transgenic rats [7]. Within astrocytes, *in vitro* exposure to HIV proteins increases expression of p16 [27, 28]. Similarly, cortical astrocytes in a humanized HIV mouse model have elevated levels of p16 compared to control animals [28]. However, astrocyte expression of p16 throughout the brain of SIV-infected NHPs had not been previously analyzed. Here, we demonstrate robust increases in astrocytic p16 with SIV infection across six brain regions implicated in HAND. These increases are also seen in the total cell population, which warrants further investigation to determine if other cell types experience similar significant increases in p16 expression with SIV infection or if this is an astrocyte-driven response. Importantly, astrocyte expression of p16 could be indicative of cellular senescence in these cells which would prevent them from performing critical homeostatic functions and lead to damaging effects in the CNS. However, p16 expression alone is not sufficient to determine a cellular senescence phenotype and future studies should assess the expression of additional molecular markers associated with senescence to better assess the status of these cells.

Another molecular marker associated with aging and an upstream regulator of the cellular senescence marker p21, is SIRT1 an NAD-dependent deacetylase. SIRT1 acts largely in an anti-aging capacity, as it inhibits several pathways associated with aging and is downregulated with age in several tissues [29]. However, the downregulation of SIRT1 expression is not consistent across all tissues, suggesting a tissue-specific alteration with age. Within the brain, SIRT1 expression has been shown to increase with age across several brain regions of Wistar rats while it is the activity of SIRT1 that is decreased [30]. We also observed an increase in SIRT1 expression with age in the temporal lobe of NHPs [13], but have not yet assessed SIRT1 activity. In the context of HIV, SIRT1 expression is decreased in the gut of SIV-infected rhesus macaques [20] and in macrophages/microglia of NHPs with SIV-encephalitis [31]. As the HIV-1 Tat protein is known to directly interact with SIRT1 and inhibit its activity [19], a decrease in expression combined with a decrease in activity could lead to drastic increases in aging processes. However, in the present study, we found both total SIRT1 expression and astrocytic SIRT1 expression to be increased with SIV infection across several brain regions (Fig. 4). It is possible these increases in expression are combined with decreases in activity as seen with normal aging in Wistar rats [30] and may even represent a compensatory mechanism to counteract the inhibition in activity brought on by Tat.

While cART treatment has been shown to reduce molecular markers of aging from that seen in chronically infected, untreated individuals in peripheral tissues [3–5], to our knowledge the impact of cART on biological aging in the brain has not previously been reported. Here, we found that p16 levels in cART treated animals only differed from that seen in chronically SIV-infected, untreated animals in a couple brain regions. In the hippocampus, we observed significant decreases in the %p16 + cells amongst cART treated animals relative to chronically infected, untreated animals that resembled levels seen in acutely infected animals (Fig. 3b). However, these levels of p16 were still significantly higher than that seen in naïve animals, suggesting that initiation of cART may be able to slow the aging process in the hippocampus but not return it to normal. Conversely, we saw an increase in the %p16 + astrocytes in cART treated animals compared to chronically infected, untreated animals in the putamen, but this may be due to a limited sample size for putamen from chronic SIV animals and warrants further investigation. For SIRT1, we saw a similar effect in the hippocampus, with expression levels being significantly reduced from that seen in chronically infected, untreated animals (Fig. 4). Together, these data suggest that cART may be important for limiting SIV-induced aging in the hippocampus which could have important implications for PLWH. It is also important to note that neurodegeneration in cART-treated animals was not significantly different from naïve animals in any brain region and was reduced from that seen in chronically infected, untreated animals in the frontal lobe, suggesting cART is protective against SIV-induced neurodegeneration.

These findings may hold clinical relevance for PLWH. Human imaging studies on brain aging in PLWH have demonstrated premature, accentuated, or accelerated aging throughout regions of the brain implicated by the cognitive and motor deficits seen in HAND, including the frontal cortex, caudate, putamen, hippocampus, thalamus, and cerebellum [14, 17, 32–42]. Importantly, cognitive decline also shows premature onset or accelerated progression in PLWH [43, 44] and post-mortem analyses demonstrate age-related epigenetic alterations in CNS tissues [6, 45, 46]. While we did not conduct brain imaging or cognitive assessments on the animals in this study, previous studies in non-human primates have demonstrated the clinical relevance of SIV-infected monkeys in HAND research. For example, studies using imaging techniques similar to those used in humans have also identified brain alterations in SIV infected rhesus macaques across brain regions implicated in HAND [15, 18, 47–53]. Additionally, SIV-infected rhesus macaques recapitulate the cognitive impairments seen in HAND [54–59].

## Conclusion

Overall, our data demonstrates for the first time that multiple markers of aging are significantly elevated with SIV infection across several brain regions implicated in HAND. Additionally, these alterations are not only correlated with neurodegeneration, but are also predictive of neurodegeneration in the frontal lobe and hippocampus suggesting a causative role. Given the importance of these brain regions for learning/memory and executive functioning, two cognitive domains that have been shown to be significantly more impaired in PLWH in the cART era than pre-cART [60], our results may have critical implications in the treatment or prevention of HAND. More work is needed to determine the direct effects of these aging markers on the progression of neurodegeneration and development of HAND and the underlying mechanisms at play.
